# Binding of established antinuclear antibodies to neurons depends on tissue fixation and underlying autoantigens

**DOI:** 10.3389/fimmu.2025.1674907

**Published:** 2025-10-10

**Authors:** Lucie Y. Li, Markus Höltje, Helle Foverskov Rasmussen, Lennard Halle, Marie Mayrhofer, Martin Blüthner, Harald Prüss

**Affiliations:** ^1^ Department of Neurology and Experimental Neurology, Charité Universitätsmedizin Berlin, Berlin, Germany; ^2^ German Center for Neurodegenerative Diseases (DZNE) Berlin, Berlin, Germany; ^3^ Berlin Institute of Health at Charité – Universitätsmedizin Berlin, BIH Biomedical Innovation Academy, BIH Charité Junior Clinician Scientist Program, Berlin, Germany; ^4^ Institute of Integrative Neuroanatomy Berlin, Charité-Universitätsmedizin Berlin, Berlin, Germany; ^5^ Department of Computational Health, Institute of Computational Biology, Helmholtz Center Munich, Munich, Germany; ^6^ Department of Autoimmune Diagnostics, Medizinisches Versorgungszentrum (MVZ) Laboratory PD Dr. Volkmann & Colleagues SE & Co. eGbR, Karlsruhe, Germany

**Keywords:** autoimmunity, ANA, HEp-2, anti-neuronal antibodies, immunofluorescence

## Abstract

Antinuclear antibodies (ANAs) are central biomarkers in rheumatological conditions and can drive disease pathology. Much less is known about the role of ANAs in neurological symptoms, although a number of experimental studies have demonstrated direct effects on neuronal function, for example in neuropsychiatric lupus erythematosus. Moreover, it is unclear whether the ANAs detected in HEp-2 cell-based assays, the gold standard for ANA diagnostics, can also be recognized in modern screening assays for anti-neuronal autoimmunity, such as staining on rodent brain sections or neuronal cultures. In this study, we therefore conducted a comparative mapping of ANA-positive sera with well-characterized HEp-2 patterns to central nervous system (CNS) tissue, utilizing fixed and unfixed murine brain sections and primary murine neurons. We screened 74 ANA-positive sera classified into 14 individual patterns and combinations thereof. Majority of the samples reacted with fixed primary neurons (99%, 73/74 sera), followed by fixed brain sections (93%, 69/74), but much less to unfixed mouse brain (54%, 40/74). While the PM/SCL- and RPOI-positive sera showed no binding to unfixed brain sections, the U1RNP (U1 nuclear ribonucleoprotein particle) and FBLN (fibrillarin) ANAs reacted strongly across all assays, indicating differences in antigen accessibility. These findings suggest that the majority of ANAs can interact with neural components, which may obscure the detection of other anti-neuronal autoantibodies. The foundational mapping of ANA binding in CNS tissue provided here can also facilitate recognition of “CNS-specific ANAs,” which bind to neuronal autoantigens but not to HEp-2 cells. Future studies should explore the association with certain neurological manifestations and the role of ANAs in neuronal pathology.

## Introduction

Neurological symptoms frequently occur in patients with rheumatological diseases. For example, in systemic lupus erythematosus (SLE), neuropsychiatric symptoms have been reported in approximately 50% of patients (1). Experimental work has demonstrated that antinuclear antibodies (ANAs) can exert pathogenic effects on neuronal tissue. A subset of anti-dsDNA (double-stranded DNA) antibodies bound the *N*-methyl-d-aspartate receptor (NMDAR) on neurons, thereby driving neuronal cell death and neuropsychiatric lupus (2).

ANAs are key biomarkers for disease definition and diagnostics in rheumatological diseases (3, 4). For example, anti-dsDNA and anti-SMAG (Smith antigen) antibodies are part of the classification criteria of SLE, as well as anti-U1RNP (U1 nuclear ribonucleoprotein particle) for mixed connective tissue disease (5). However, not much is known about their role in neurological conditions. Although a number of studies have reported higher frequencies of HEp-2 ANAs in patients with multiple sclerosis (6) and neuromyelitis optica spectrum disorder (NMOSD) (7), potentially pointing to worse clinical outcomes (8), it has not been systematically assessed which ANA subtypes can also bind to neuronal tissue and cause pathology. At the same time, ANAs are increasingly detected in screening assays for anti-neuronal autoantibodies, including tissue-based assays with rodent brain sections (9), where they may obscure the detection of disease-specific autoantibodies as ANAs cross-reacting with neuronal structures could lead to the misinterpretation of diagnostic assays for neuronal antibodies. Due to the lack of an image catalogue of ANA binding to neuronal cells, it is difficult to determine whether the underlying ANAs show a comparable histology pattern seen on HEp-2 cells, the gold standard for ANA routine diagnostics. Alternatively, the pattern on brain sections may represent central nervous system “(CNS)-specific ANAs,” referring to a group of intracellularly binding autoantibodies that bind to neurons but are not detectable on HEp-2 cells (10).

## Methods

### Patients and samples

ANA-positive samples from 74 patients and one control serum were selected from our collection of anonymized leftover samples. The selection criteria were monospecificity for one antigen, clear ANA indirect immunofluorescence test (IFT) patterns, and a clinically relevant titer.

### Staining of serum samples on HEp-2 cells

HEp-2 staining was performed strictly according to the protocol supplied with the kit on a QUANTA-Lyser 4000 QL4K (Inova/Werfen, Bedford, MA, USA) using the NovaLite ANA Kit (ref. no. 704320; Inova/Werfen). Sera were routinely diluted 1:80 and incubated on 12-well glass slides coated with HEp-2 cells, which were already fixed and permeabilized by the manufacturer. Bound serum antibodies were detected with a ready-to-use solution of anti-human IgG (fluorescein isothiocyanate, FITC) in conjunction with DAPI to enable automated focusing. Titrations were performed by geometrical serial dilutions of the serum samples. The titers and staining patterns were assessed on-screen using the QUANTA-Link software (Inova/Werfen) on a calibrated monitor supported by visual inspection.

### Identification and quantification of specific antibodies

Specific antigen identification was conducted with ELISA using kits from Thermo Fisher/Phadia (Waltham, MA, USA) [U1RNP (ref. no. 14-5501-01), CENP-B (ref. no. 14-5505-01), SMAG (ref. no. 14-5672-01), RO-52 (ref. no. 14-5598-01), RO-60 (ref. no. 14-5525-01), SSB (ref. no. 14-5504-01), SCL (ref. no. 14-5637-01), PM/SCL (ref. no. 14-5602-01), RIBO (ref. no. 14-5521-01), FBLN (ref. no. 14-5605-01), and RPOI (ref. no. 14-5599-01)] on ImmunoCAP250 PHADIA Prime version 2.3.11 or kits from Euroimmun (Lübeck, Germany) [ssDNS (ref. no. EA 1576–9601 G), DFS70 (ref. no. EA 159z-9601G), HIAK (ref. no. EA 1560–9601 G), and NUCLEO (ref. no. EA1574–9601 G)] on EUROLab Workstation ELISA 45 version 2.6.197 strictly following the manufacturers’ instructions. The results were interpreted according to the manufacturer’s cutoff.

Antibodies against dsDNA were detected with the Farr assay using a kit from IBL (ref. no. RE19011; Hamburg, Germany) following the manufacturer’s instructions and were measured on a Gamma-Counter RA-107, PerkinElmer Wizard (Waltham, MA, USA). All results ≥7 IU/ml were considered positive, while results <7 IU/ml were considered negative. Antibodies against NOR90 and proliferating cell nuclear antigen (PCNA) were detected using Western blot and radioimmunoassay respectively, according to the manufacturer’s protocol. For detailed protocols, please refer to the **Supplementary Material**.

### Staining of primary neuronal cultures

E16–17 embryos were obtained from pregnant Swiss mice sacrificed by cervical dislocation. The embryonic hippocampi and partial cortices were isolated and collected in nutrient broth (NB) medium containing 10 ml B27, 5 ml penicillin/streptomycin, 1.25 ml l-glutamine, and 485 ml neurobasal medium. Thereafter, an N-medium (50 ml fetal calf serum, 5 ml penicillin/streptomycin, 5 ml l-glutamine, 10 mM HEPES, 1 mg/ml insulin, 44 mM glucose, and 5 ml collagen G, filled up to 500ml with Dulbecco’s modified Eagle’s medium) was added and the tissue centrifuged at 800 rpm for 2 min at 4°C. The pellet was resuspended in N-medium without collagen, centrifuged again at 800 rpm for 2 min at 4°C, and the cells diluted in NB starter medium [25 µl Na-glutamate (100 mM)/100 ml NB medium). Coverslips in 24-well plates were incubated with poly-l-lysine solution 1:20 in phosphate-buffered saline (PBS) overnight and then coated with N-medium containing collagen. Finally, the medium was removed and the cell solution added (8 × 10^4^/ml). The cells were incubated for 10–14 days at 37°C and then used for immunostaining.

For staining, the cells were washed with 10% PBS two times and fixed with 10% paraformaldehyde (PFA) for 10 min. Consecutively, neuronal cells were incubated with patient serum diluted 1:200 overnight, followed by incubation with a secondary antibody. For IgG detection, sections were incubated with Alexa Fluor 488 coupled with anti-human IgG (cat. 109-545-003; Dianova, Hamburg, Germany).

### Staining on fixed and unfixed murine brain sections

For tissue sections of unfixed mouse brains, the animals were sacrificed and the brains were removed and snap-frozen in −50°C cold 2-methyl butane. Fixed brain sections were obtained transcardially from animals perfused with 4% PFA. Sagittal sections (20 µm) were cut and then stained with patient sera at 1:200 dilution, in line with laboratory routine testing procedures. For IgG detection, the sections were incubated with Alexa Fluor 488 coupled with anti-human IgG (cat. 109-545-003; Dianova).

Images were taken at ×40 magnification using a Leica DMLB epifluorescence microscope or a Leica SP2 confocal imaging system. Image analysis was conducted using ImageJ version 1.54f and Adobe Photoshop 22.2.0 software. Descriptive statistics were analyzed in GraphPad Prism (version 9).

## Results

We selected ANA-positive sera from 74 patients with 14 individual HEp-2 cell-classified staining patterns and combinations thereof, as well as one control serum negative on HEp-2 cells. The samples were tested for reactivity against CNS epitopes using primary murine neurons and PFA-fixed and unfixed murine brain sections. All sera showed >1:80 binding to HEp-2 cells and were analyzed for binding against specific antigens. The samples covered all subgroups with the most established ANA antigens, including dsDNA, ssDNA (single-stranded DNA), U1RNP (U1 nuclear ribonucleoprotein particle), CENP-B (centromere protein B), RO-52 (Ro/SS-A-p52), RO-62 (Ro/SSA-p60), SSB (La/SS-B), SCL (Scl70/topoisomerase), PM/SCL (polymyositis/scleroderma), DFS70 (dense fine speckles), HIAK (histone), NUCLEO (nucleosome), FBLN (fibrillarin), and RPOI (RNA polymerase I–III). Some sera contained ANAs reactive to more than one antigen (15/74), mostly belonging to the same particle subgroup (the hY-RNP subgroup including RO-52, RO-60, and SSB or the chromatin subgroup including HIAK, NUCLEO, and dsDNA).

**Figure 1:**
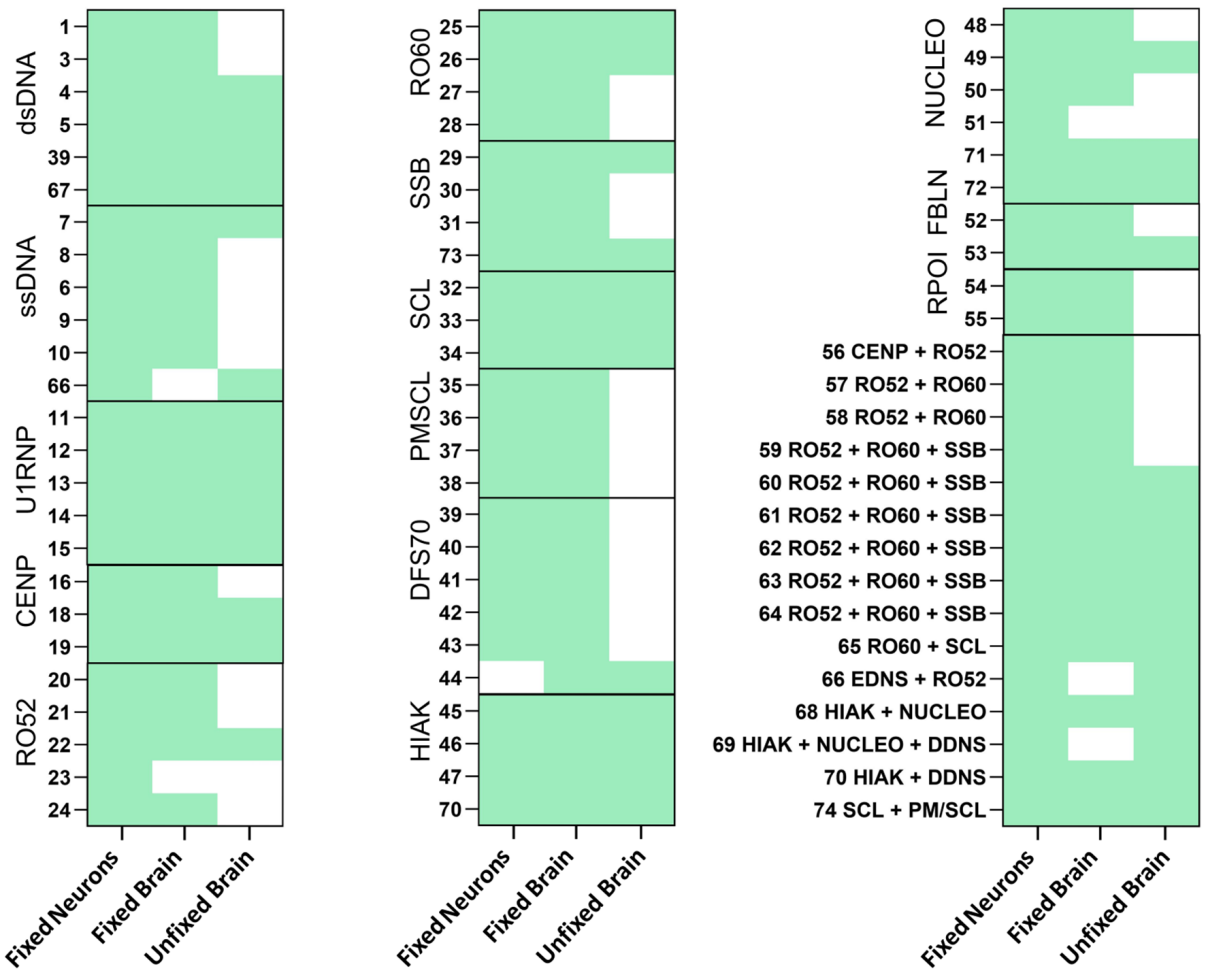
Reactivity of antinuclear antibody (ANA)-positive patient sera on fixed neurons and both fixed and unfixed brain sections. ANA-positive sera with different antigens were stained in a dilution of 1:200 on fixed neurons (E16-17) and on both fixed and unfixed murine brain sections. Bound immunoglobulin G (IgG) was detected using Alexa Fluor 488-labeled secondary anti-human IgG antibody. Reactivity for each assay is indicated in *green*.

Comparisons between the different assays revealed marked differences (**Figure 1**), with the overall highest rate of reactivity in fixed primary hippocampal neurons, where 73 of 74 HEp-2-positive sera showed an immunofluorescence signal. The sera were in large part also positive on fixed murine brain sections (93%, 69/74). On unfixed murine brain sections, only approximately half (54%, 40/74) of the sera were reactive. Clear differences in the ANA antigen subgroups regarding reactivity to unfixed tissue were observed. In the PM/SCL group and the RPOI group, none of the tested sera (0/5 and 0/2, respectively) showed binding to unfixed tissue. In contrast, sera of the U1RNP, HIAK, and SCL subgroups were all reactive on unfixed murine brain tissue, although the small sample sizes prevent generalizability and require confirmation in larger sample cohorts.

**Figure 2:**
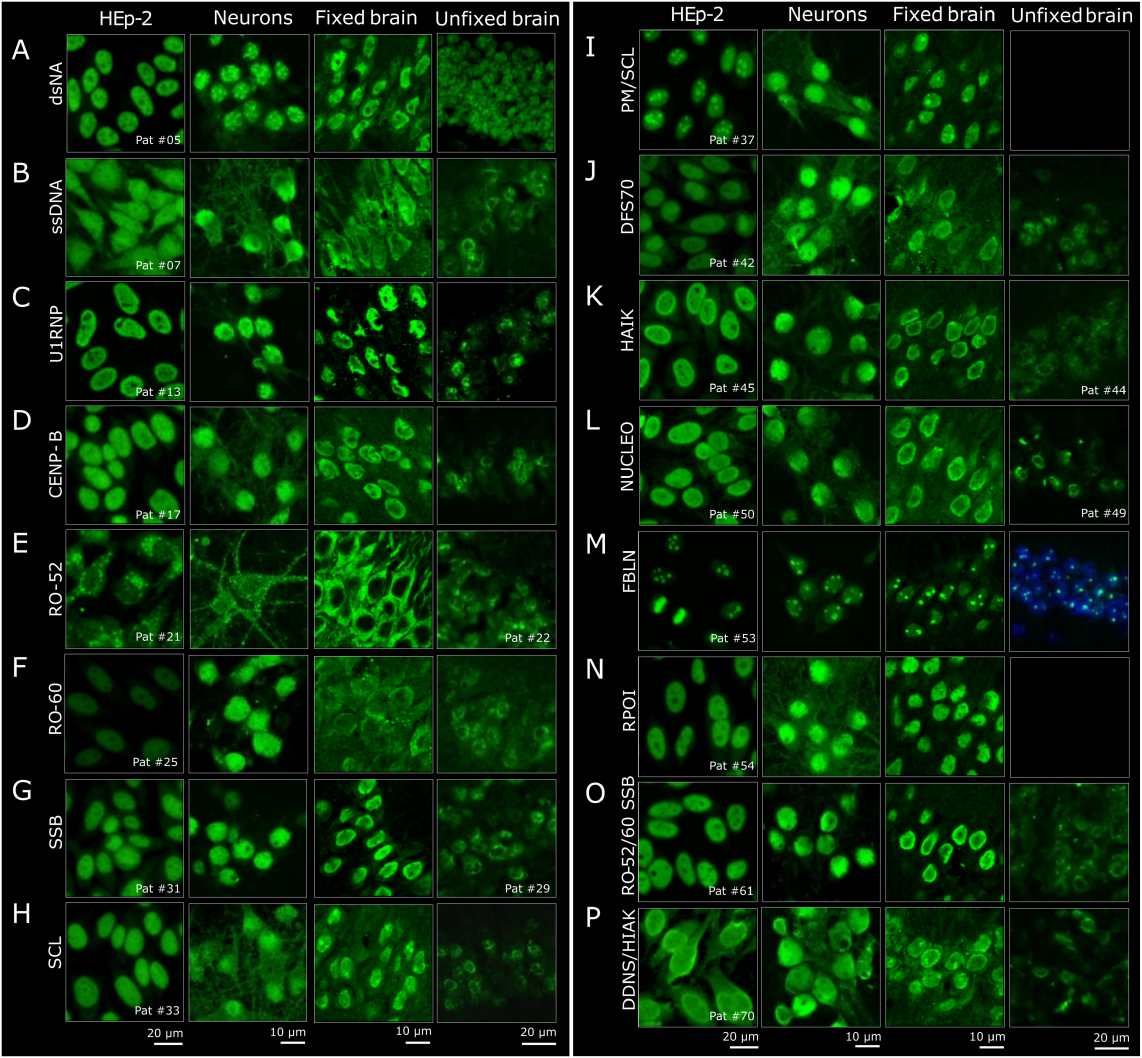
Histological comparison of the staining patterns of patient sera on HEp-2 cells and central nervous system (CNS) tissue. **(A–P)** The staining patterns of HEp2-reactive patient sera (*first column*) were compared to the staining on primary neurons (*second column*) and on fixed and unfixed brain sections (*third* and *fourth columns*). For the brain sections, representative regions from the hippocampus are shown. When available, one reactive serum per group is depicted. In the *last two rows* (sample no. 61 and no. 70), examples of two sera with combined reactivity against multiple antigens are shown.

Detailed analysis of the staining patterns across neuronal assays and comparison to standard HEp-2 patterns showed a number of differences at the microscopic level (**Figures 2A–P**). The characteristic nuclear HEp-2 pattern of majority of the samples was closely reflected also on cultured mouse neurons (**Figure 2**, second column), for example, with the U1RNP (**Figure 2C**), SSB (**Figure 2G**), DFS70 (**Figure 2J**), HIAK (**Figure 2K**), or NUCLEO antibodies (**Figure 2L**). Similarly, the samples with multiple ANAs had corresponding staining patterns, such as serum no. 61 (**Figure 2O**) with a nuclear and speckled staining and serum no. 70 (**Figure 2P**) with a nuclear and homogeneous staining on both HEp-2 cells and cultured neurons. In the case of the dsDNA- (**Figure 2A**) and ssDNA-reactive ANAs (**Figure 2B**), the nuclear and speckled patterns were comparable, although the speckles appeared more contrasted on the neurons for dsDNA and more homogenous for ssDNA compared with HEp-2 cells. The cytoplasmic Golgi-like staining of the RO-52 pattern (**Figure 2E**) was equally seen on neurons and HEp-2 cells. As RO-52 ANAs rarely stain HEp-2 cells and show a cytoplasmic Golgi-like pattern (resembling AC-22), serum no. 21 likely contained additional antibodies, e.g., against Golgi antigens. In the ANA subgroups RO-60 (**Figure 2F**), CENP-B (**Figure 2D**), SCL (**Figure 2H**), PM-SCL (**Figure 2I**), and RPOI (**Figure 2N**), the neuronal staining exceeded the HEp-2 nuclear staining and included parts of the cytoplasm in a fibrillary pattern.

The immunofluorescence staining on PFA-fixed murine brain sections (**Figure 2**, third column) was next compared to HEp-2 cells. For better comparison with the primary mouse neurons derived from the hippocampus, the staining patterns were evaluated in the hippocampus of murine brain sections as well. This comparison showed equivalent findings, in particular when the HEp-2 pattern was nuclear, such as with U1RNP, DFS70, and RPOI (**Figures 2C, J, N**). Sera containing ANAs against SSB, HIAK, and NUCLEO (**Figures 2G, K, L**) showed the expected nuclear staining, but with more emphasis on the outer rim on the PFA-fixed murine brain sections. Sera from the dsDNA and ssDNA (**Figures 2A, B**) subgroups stained the fixed brain mostly nuclear, but with a coarser pattern. The PM/SCL sera (**Figure 2I**) showed the characteristic nucleolar pattern known from HEp-2 cells (AC-8 pattern). Other samples showed differences between the two assays. For example, CENP-B (**Figure 2D**) spared the nucleoli on fixed brain compared with HEp-2 cells, and RO-60 (and even more so RO-52) showed stronger cytoplasmic staining, with RO-52 leaving out the nucleus entirely (**Figures 2E, F**). The SCL serum (no. 33) stained the cytoplasm more homogeneously than on cultured neurons, but showed intense spots, most likely representing nucleoli (**Figure 2H**). Similar nucleolus-like spots were observed only on fixed brain, with serum no. 70 containing dsDNA and HIAK ANAs, both antigens with an AC-1 pattern (**Figure 2P**). Given that the HEp-2 cell assay utilizes methanol/acetone fixation according to the majority of used commercial kits whereas PFA is used for the fixation of brain sections and neuronal cultures, differences in the staining patterns due to fixation agents cannot be fully excluded and should be addressed in future investigations.

Much less signal was detectable on unfixed mouse brain (**Figure 2**, fourth column) compared with HEp-2 cells and fixed neurons for majority of the samples, and sera with PM-SCL and RPOI ANAs showed no binding at all (**Figures 2I, N**). Only the dsDNA-positive sera appeared similar, with a relatively homogenous nuclear pattern (**Figure 2A**). In contrast, subgroups ssDNA, CENP-B, RO-52, RO-60, SSB, SCL, DFS70, HIAK, and NUCLEO (**Figures 2B, D–H, J–L**) showed less evenly distributed speckling in the nucleus, giving the impression of spared nucleoli. Such patterns were not observed on HEp-2 cells, cultured neurons, or fixed brain sections, underlining the influence of tissue preparation for the diagnostic assays.

Comparison of all four assays revealed that only individual ANAs showed comparable staining patterns. The FBLN sera presented a distinct dotted nucleolar staining across all assays and fixation methods (**Figure 2M**). Similarly, the U1RNP sera had a relatively uniform speckled nuclear pattern, although weaker on unfixed brain (**Figure 2C**). In general, the staining patterns were most comparable between HEp-2 cells and fixed cultured primary neurons, often also on fixed mouse brain, while unfixed brain demonstrated the least overlap.

## Discussion

In this study, we collected ANA-positive sera with well-characterized HEp-2 patterns and conducted a comparative mapping to the CNS tissue, utilizing fixed and unfixed murine brain sections and primary murine neurons. Of the 74 ANA-positive sera falling into 14 pattern categories, almost all samples reacted with fixed primary neurons, demonstrating comparable staining patterns. Fixed brain sections also recognized majority of the ANAs with similar patterns, despite some differences in the speckling and intensity of nuclear staining. In contrast, unfixed mouse brain reacted with only half of the well-defined ANA-positive sera, and the nuclear speckling was generally less homogeneous. As fixation preserves and stabilizes the tissue architecture, the lower reactivity of ANAs in unfixed tissue is in line with this principle. The detailed staining differences observed between the methanol/acetone-fixed HEp-2 cells and the PFA-fixed neuronal tissues likely reflect the distinct manner in which these fixation methods preserve antigenic structures and conformations.

The almost complete recognition by fixed cultured neurons suggests that the majority of ANAs can interact with neural components. This revives the “old” unanswered question of whether they can exert pathological effects on neurons leading to neurological symptoms. In AQP4 antibody-positive patients with NMOSD, ANA seropositivity was correlated with poor clinical outcomes (8). In a recent study that examined autoimmune mechanisms in psychotic syndromes, almost one-fourth of the patients were HEp-2 ANA-positive, which correlated with the MRI pathologies (11). More work has been done in rheumatological conditions, where certain ANAs were found to be associated with the occurrence of neuropsychiatric SLE (NPSLE). For example, dsDNA antibodies typically found in SLE can cross-react with the NR2 subunit of the NMDAR and exert pathogenic effects by prolongation of the excitatory synaptic transmission, thus driving excitotoxic neuronal death (2, 12). The NR2 antibody levels in the cerebrospinal fluid (CSF) were higher in patients with NPSLE compared with the non-SLE controls. Similarly, the U1RNP ANAs in the CSF were linked to NPSLE (13), and cross-reactive ANAs against ribosomal P were associated with NPSLE and caused a depression phenotype in mice (14–16). Finally, the well-known anti-Hu antibody (type 1 anti-neuronal nuclear antibody, ANNA-1), which is a biomarker of paraneoplastic neurological syndromes, has been recently shown to directly cause neuronal dysfunction (17). These studies collectively suggest that ANAs may contribute to neurological symptoms. Confirmation, however, awaits further detailed experimental and clinical research, including the generation of patient-derived monoclonal ANAs and their use in functional assays and animal models.

The mapping of the ANAs on CNS tissues presented here may provide an initial framework to explore their characteristics. First and foremost, we documented how strongly and specifically ANA reactivity depends on the fixation of the rodent tissue. While the PM/SCL antibody-positive sera demonstrated a distinct nucleolar staining pattern on HEp-2 cells and fixed tissue, they completely lacked binding to unfixed mouse brain. Thus, fixation and permeabilization appear necessary to expose the PM/SCL epitopes, which are components of the human exosome involved in the processing of 5.8S rRNA (18). The main PM/SCL target antigen presumably is a localized α-helical secondary structure within the PM/SCL-100 protein (19). Its subcellular localization within a larger exosomal protein complex likely restricts accessibility under unfixed conditions. Similarly, the RPOI antibodies directed against RNA polymerases that are ubiquitously present in tissues including the brain did not show reactivity without fixation. In contrast, the U1RNP subgroup exhibited consistent reactivity across all neuronal assays, including unfixed murine brain. This observation aligns with the highly conserved nature of the U1RNP antigen U1-70K, which is part of the small nuclear ribonucleoproteins (snRNPs) that play a central role in pre-mRNA splicing (20). Similarly, with the FBLN ANAs, the underlying antigen fibrillarin is a highly conserved protein component of the snRNP complex, and the distinct dotted nucleolar pattern was visible across all fixation and assay conditions. Our findings therefore highlight the challenges in interpreting ANA diagnostics when searching for anti-neuronal autoantibodies using different tissues. It will be interesting to determine whether a reduced background from ANA binding can facilitate the diagnostics of some neuronal targets using unfixed murine brain sections.

Throughout the literature, the term ANA has not always been used consistently, with some suggesting only using ANA for HEp-2 indirect immunofluorescence assay (IFA). Here, we refer to CNS-specific ANAs as a group of autoantibodies that bind to the neuronal nuclei that are not detectable on HEp-2 cells. Assay differences may be particularly relevant for this particular group of ANAs. CNS-specific ANAs were more prevalent in multiple sclerosis compared with NMOSD or healthy controls (10, 21), and single cases suggested that CNS-specific ANAs may contribute to neuropsychiatric abnormalities, such as progressive cognitive decline (22). This relatively new concept can drive research on novel autoantibodies targeting the nuclei of brain cells, as these are—in contrast to “normal” HEp-2 ANAs—more likely to induce neuropathology and clinical symptoms. Future work could include the immunoblotting and immunoprecipitation of brain tissue fractions combined with proteomic analyses to identify neuronal ANA targets, ideally supported by monoclonal antibodies for verification.

Taken together, the present study describes the spectrum of well-established HEp-2 ANAs binding to neuronal structures and identified important differences related to tissue fixation and underlying autoantigens. The foundational comprehensive mapping of ANA binding in CNS tissue provided here can facilitate the recognition of CNS-specific ANAs and the detection of neuronal autoantibodies that are potentially obscured by ANAs and is a starting point for estimating the potential association with clinical symptoms. While the current study focused on the histological comparison of different ANA patterns, future work should also attempt to correlate these ANA patterns and titers with neuropsychiatric symptoms. Future studies should explore in detail the association with certain neurological manifestations, but should also utilize patient-derived monoclonal antibodies for functional assays and *in vivo* models in order to understand the role of ANAs in neuronal pathology.

## Data Availability

The raw data supporting the conclusions of this article will be made available by the authors, without undue reservation.

## Glossary

dsDNA: double-stranded DNA
ssDNA: single-stranded DNA
U1RNP: U1 nuclear ribonucleoprotein particle
CENPB: centromere protein B
SMAG: Smith antigen
RO52: Ro/SS-A-p52 (Sjörgen’s syndrome-related antigen A)
RO62: Ro/SS-A-p60 (Sjörgen’s syndrome-related antigen A)
SSB: La/SS-B (Sjörgen’s syndrome-related antigen B)
SCL: Scl-70/topoisomerase I (scleroderma, extractable 70-kDa fragment)
PM/SCL: PM/Scl (polymyositis/scleroderma)
DFS70: DFS70/LEDGF, dense fine speckles/lens epithelium-derived growth factor
HIAK: histone
NUCLEO: nucleosome
PCNA: cyclin, auxiliary protein of DNA polymerase delta
RIBO: ribosomal P proteins
FBLN: fibrillarin
RPOI: RNA polymerase I–III
NOR90: NOR-90/human upstream binding factor 1 (hUBF-1)
