# Supplementary material for: Binding of established antinuclear antibodies to neurons depends on tissue fixation and underlying autoantigens
Source: Front Immunol. 2025 Oct 10;16:1674907. doi: 10.3389/fimmu.2025.1674907 (PMC12549672; doi:10.3389/fimmu.2025.1674907)
Supplement: Supplementary file 1 [file Table1.docx]

Supplementary Material

# Supplementary Methods

Antibodies against NOR90 were detected via western blot following manufacturers protocol. Briefly, patient sera were diluted 1:200 in dilution buffer and incubated on PVDF membranes for 90 min at RT with steady agitation. Membranes were washed 3x for 5 min each with washing buffer, incubated with1000 μl AP-anti-human-IgG/M-Konjugate for 60 min at RT, washed 3x for 5 min each, incubated with 1000 μl ready-to-use AP-Substrate for 5 min at RT. Substrate solution was discarded and reaction stopped by adding 1000 µl washing buffer followed by washing 3x for 5 min each. For analysis PVDF membranes were dried at 45-50°C for 5-10 min. PVDF membranes with a band at 88-90 kDa were regarded as positive.

Antibodies against PCNA were detected via radioimmuno assay according to manufacturer´s protocol. Briefly, in vitro transcription/translation (ivTT) products radiolabelled with 35S-methionine-PCNA were diluted in dilution buffer (20 mM Tris-HCl / 150 mM NaCl / 0,15 % Tween-20 / 100000 KIE Aprotinin (Bayer, Leverkusen) / 10 mM Benzamidine / 0,1 % BSA) to an activity of 40,000 cpm in 50 µL. 5 µL serum samples were pipetted into a 96-well plate and 50 µL diluted ivTT-PCNA were added to each well, incubated for 3 h at 4° C on a plate shaker, 20 µL recombinant Protein A-Sepharose (Cytiva, Freiburg) were added to each well and incubated for 1 hour at 4° C. The reaction mixes were transferred to a 0,65 µm Durapore 96-well filter plate (Millipore, Schwalbach). Unbound antigen was removed by vacuum filtration and washing of the filter plates 30 times with 100 µL washing buffer (20 mMTris-HCl / 150 mMNaCl/ 0,05 % Tween-20) each. Plates were dried overnight, 20 µL liquid scintillation mix MicroScintO (PerkinElmer, Rodgau) were added to each well and the plates were counted using a Topcount NXT 96-well liquid scintillation counter (PerkinElmer, Rodgau).

Data was evaluated according to A. Frey et al. (Frey et al., 1998). All results > 15 were considered positive, results between 10 and 15 are located in a grey zone.
